# Prevalence, Associations and Comorbidity of Cannabis Use and Cannabis Use Disorders in the Australian National Mental Health Surveys From 2007 to 2020–22

**DOI:** 10.1111/dar.70134

**Published:** 2026-03-08

**Authors:** Jack Wilson, Matthew Sunderland, Siobhan O’Dean, Tim Slade, Danielle Dawson, Olivia Dobson, Janni Leung, Gary Chan, Maree Teesson, Wayne Hall, Nicola Newton, Valentina Lorenzetti, Emily Stockings

**Affiliations:** ^1^ The Matilda Centre for Research in Mental Health and Substance Use The University of Sydney Sydney Australia; ^2^ National Centre for Youth Substance Use Research, School of Psychology The University of Queensland Brisbane Australia; ^3^ School of Psychology The University of Queensland Brisbane Australia; ^4^ Neuroscience of Addiction and Mental Health Program, Healthy Brain and Mind Research Centre, School of Behavioural and Health Sciences, Faculty of Health Sciences Australian Catholic University Melbourne Australia

**Keywords:** cannabis, cannabis use disorder, comorbidity, epidemiology

## Abstract

**Introduction:**

As cannabis policies become more liberal, and products increasingly potent, it is important to monitor changes in rates of cannabis use and those at risk of harm. We used data from the largest national mental health survey in Australia to examine changes in prevalence, associations and comorbidity of past 12‐month cannabis use and cannabis use disorder (CUD) from 2007 to 2020–22.

**Methods:**

Data were drawn from the two most recent Australian National Surveys of Mental Health and Wellbeing (age range: 16–85): 2007 (*n* = 8841) and 2020–22 (*n* = 15,893). The prevalence of use and CUD were estimated and logistic regression was used to examine associations between individual characteristics and cannabis use outcomes, as well as changes in strength of association over time.

**Results:**

Prevalence of recent cannabis use was stable from 2007 (6.7%, 95% CI 6.0%, 7.3%) to 2020–22 (6.7%, 95% CI 6.2%, 7.1%), as was CUD (1.0%, 95% CI 0.7%, 1.3%; 0.6%, 95% CI 0.4%, 0.8%). Respondents were significantly more likely to report CUD if they were younger (16–25 years), male, initiated cannabis use < 18 years, reported recent polysubstance use, exhibited other substance and mental disorders and had visited mental health services (OR 3.26–78.00). Compared to 2007, the association between younger age and CUD was stronger in 2020–22 (OR 2.39, 95% CI 1.20, 4.77), whereas the association between polysubstance use and CUD was weaker (OR 0.42, 95% CI 0.20, 0.91).

**Discussion and Conclusions:**

Findings suggest that population‐level use and CUD may have remained stable over time, but young people may now be more vulnerable to developing CUD.

## Key Point Summary

- In 2020–22, the prevalence of past 12‐month cannabis use and cannabis use disorder (CUD) among Australians was 6.7% and 0.6%, respectively.
- Compared to 2007, the prevalence of use and CUD remained stable.
- The relationship between younger age and CUD became stronger from 2007 to 2020–22, whereas the association between polysubstance use and CUD weakened.

## 1 Introduction

In 2022, approximately 228 million people used cannabis globally, making it one of the most commonly used drugs worldwide [1]. According to regional estimates, Australia is one of the leading cannabis consumers among general (11.5%) and youth (14–17 years; 13.3%) populations [2]. Australians also exhibit some of the highest rates of cannabis dependence and related harms [1, 2]. As cannabis products have become increasingly accessible in Australia [2, 3], it is increasingly important to monitor patterns of cannabis use in nationally representative samples and identify those most at risk of experiencing harms.

International data suggests a rise in the prevalence of cannabis use in regions with more liberal cannabis policies. Medical cannabis was first introduced formally at a state level in the United States (US) in 1996 (California), with non‐medical legalisation following in 2012 (Washington) [4]. Since then, there has been a nation‐wide increase in the prevalence of cannabis use and cannabis use disorder (CUD) [5, 6], particularly among certain population groups, notably among those aged 65+ [7], males [4] and persons with existing mental health disorders [8, 9]. Meanwhile, a difference‐in‐difference analysis suggested that US states that enacted non‐medical and medical cannabis laws experienced a respective 25% and 5% increase in any annual use among adults [10]. Similar increases in annual use have been observed among adults in Canada following its legalisation of non‐medical use in 2018 [11].

Australia's regulatory cannabis framework is now at a stage largely comparable to the US 20 years ago. Medicinal cannabis was legalised federally in Australia in 2016 allowing persons with specific medical conditions to access cannabis through a prescription pathway via the Australian Therapeutic Goods Administration [12]. Early indicators of use indicate a substantial rise in the number of medical cannabis approvals since 2016, with more than 1.4 million approvals from July 2016–December 2023, equating to approximately 300,000 to 500,000 Australians (due to likely multiple approvals per individual) [13]. In fact, growing concerns over safety and unregulated access to medical cannabis in Australia recently led to the Therapeutic Goods Administration undertaking a consultation process to address such issues [14]. Like the US, legal recreational use is less common, with only the Australian Capital Territory having legalised the possession and cultivation of a limited quantity of cannabis for personal recreational use in 2020 [15]. Given this growing momentum in legal avenues for cannabis use, Australia may also be witnessing a change in the prevalence, associations and comorbidity of cannabis use. In conjunction, the amount of tetrahydrocannabinol (THC) in illicit cannabis markets has substantially increased over the past 50 years, resulting in more potent products [3, 16]. There is now some evidence that consumers of high potency cannabis are at a greater risk of exhibiting symptoms of psychosis and CUD [17]. This combination of increased access to higher potency cannabis calls for close monitoring of national trends in cannabis use and CUD in Australia.

The National Survey of Mental Health and Wellbeing (NSMHWB) is Australia's largest and most comprehensive household data source on mental and substance use disorders. Unlike Australia's National Drug Strategy Household survey, the NSMHWB administers the modified version of the World Mental Health Composite International Diagnostic Interview (WHM‐CIDI 3.0; [18]) and thus collects data on diagnostic criteria for substance use disorders in accordance with the Diagnostic and Statistical Manual of Mental Disorders, 4th edition (DSM‐IV). It is thus the best national indicator of CUD available in Australia. The two most recent waves of the NSMHWB were conducted in 2007 [19], and 2020–22, a period in which major cannabis reforms were introduced. This provides an unparalleled opportunity to examine prevalence and trends of cannabis use and individual characteristics at the population level. The current study aims to assess changes in the prevalence, associations and comorbidity of cannabis use and CUD in Australia from 2007 to 2020–22 by:

1. Examine changes in prevalence of past 12‐month cannabis use and CUD between 2007 and 2020–22;
2. Establish the association between demographic, clinical and health characteristics and past 12‐month cannabis use and CUD for the combined 2007 and 2020–22 sample;
3. Examine changes in the association between demographic, clinical and health characteristics and past 12‐month cannabis use and CUD between 2007 and 2020–22.

## 2 Methods

### 2.1 Sample

Data were drawn from the 2007 (*n* = 8841; 60% response rate) [20] and 2020–22 (*n* = 15,893; 52% response rate) NSMHWB, cross‐sectional surveys funded by the Australian Government Department of Health and conducted by the Australian Bureau of Statistics (ABS). Due to interruptions caused by the COVID‐19 pandemic, the 2020–22 survey was collected in two cohorts, the first between December 2020 and July 2021 (*n* = 5554) and the second between December 2021 and October 2022 (*n* = 10,339) [21]. These samples have since been combined into a single dataset with appropriate demographic weights to align with the estimated residential population of Australia (e.g., educational attainment, labour force status). Respondents were usual residents in Australia aged 16–85 years living in private dwellings within urban and rural areas across all states and territories. People in very remote areas as well as discrete Aboriginal and Torres Strait Islander communities were excluded. Slade et al. [21] provide further details of the methodology. The use of the data was approved by the Australian Bureau of Statistics in line with their comprehensive data use and safety rules. Given this is a secondary analysis of publicly available data the current study was exempt from human ethics review.

### 2.2 Measures

Most of the data was collected via face‐to‐face surveys with an ABS Interviewer. During collection of the second cohort for the 2020–22 survey, 446 households (4.3% of Cohort 2 fully responding households) completed the survey via video call with an ABS Interviewer. The surveys collected the following information: demographics, household details, socioeconomic characteristics, general health and wellbeing, mental disorders, substance use, suicidality, self‐harm, disordered eating and use of health and social support services. Further detail is subsequently provided for the data used in the current analysis.

#### 2.2.1 Substance Use and Substance Use Disorders

Participants were asked whether they had ever used cannabis, tobacco, non‐medical prescription drugs, stimulants, sedatives and opioids. Those that indicated that they had used any of these drugs at least five times in their life were asked more detailed questions regarding characteristics of substance use and diagnosis of substance use disorder. Those using cannabis at least five times in their life were asked at what age they first used, whether they had used in the past 12 months, their frequency of use in the past 12 months and the amount used on a typical day in the past 12 months. Diagnostic criteria for any DSM‐IV cannabis or other substance use disorder (abuse or dependence) were assessed using the WHM‐CIDI 3.0; [18]. This gold standard diagnostic instrument has demonstrated strong psychometric properties and has been calibrated against the structured clinical interview for DSM‐IV (SCID‐IV; [22]).

#### 2.2.2 Mental Health Disorders

The WHM‐CIDI 3.0 also assessed past 12‐month diagnoses of DSM‐IV major mood disorders (i.e., major depressive disorder, dysthymia and bipolar disorder), anxiety disorder, social anxiety disorder, obsessive compulsive disorder and post‐traumatic stress disorder.

#### 2.2.3 Demographics

Participants were asked to indicate their age, sex at birth, labour force status, highest level of education achieved (i.e., did not complete school, completed school only, post‐school qualifications), country of birth, residential area (i.e., major cities, inner regional, outer‐regional, remote or very remote) and socioeconomic disadvantage (i.e., SEIFA; Index of Relative Socioeconomic Disadvantage; where low score indicates high disadvantage) at time of the survey. From 2007 to 2020–22, the demographic and socio‐economic modules were updated to align with current ABS standards.

#### 2.2.4 Health Service Use

Service use was assessed by asking participants whether they had a consultation with a general practitioner, psychiatrist, psychologist, or other health professional for their mental health, which may include problems with substance use, in the past 12 months.

### 2.3 Statistical Analysis

Population weights were applied to ensure that the sample resembled the sex and age distribution in the population. Prevalence of past 12‐month cannabis use and any CUD in 2007 and 2020–22 was calculated, along with frequency and quantity of use among people who use cannabis and reported a CUD. To examine the degree of association between demographic, clinical and health characteristics and cannabis use outcomes, separate univariable binary logistic regressions were conducted among the combined sample (2007 and 2020–22). Effect size estimates were presented as odds ratios (OR) and 95% confidence intervals (CI) for independent variables that were significantly associated with the outcome at a *p*‐value of < 0.05. To investigate whether the relationship between individual characteristics and cannabis use outcomes has changed over time, year of follow‐up (2007 vs. 2020–22) was included as an interaction term. In this case, an OR greater than 1 would represent a strengthening of the relationship between an individual characteristic and past 12‐month cannabis use/CUD from 2007 to 2020–22. Significant interactions at *p* < 0.05 were plotted using predicted probabilities of past 12‐month cannabis use/CUD. If cell counts did not exceed *n* = 10, variables were either removed from analysis or categories were collapsed as per ABS data safety rules. All analyses were conducted in the ABS dataLab, for which the lead author had authorised access and had completed all necessary training.

## 3 Results

### 3.1 Frequency and Quantity of Use

#### 3.1.1 Past 12‐Month Cannabis Use

The prevalence of past 12‐month cannabis use in 2020–22 was 6.7% (95% CI 6.2%, 7.1%; equivalent to 1,320,437 Australians), which was stable from 2007 (6.7% 95% CI 6.0%, 7.3%; Table 1). Among those reporting cannabis use in 2020–22, the majority reported consuming cannabis less than once a week (68.3%, 95% CI 64.3%, 72.0%), with the remaining respondents equally split between using cannabis 1–4 days per week (17%, 95% CI 14.2%, 20.4%) and 4–7 days per week (14.7%, 95% CI 12.0%, 17.9%). This distribution of frequency of use was similar between 2007 and 2020–22, with overlapping CIs. An average of 2.60 g (95% CI 2.30, 2.91) and a median of 1 g (interquartile range [IQR] 1–2) of cannabis were consumed on a typical day in 2020–22. The amount of cannabis was unchanged between 2007 and 2020–22.

**TABLE 1:** Prevalence and characteristics of past 12‐month cannabis use and DSM‐IV any cannabis use disorder in Australians aged 16–85.

	Past 12‐month cannabis use		Past 12‐month DSM‐IV any cannabis use disorder	
	2007	2022–23	2007	2023
Total (%), 95% CI	6.7 (6.0, 7.3)	6.7 (6.2, 7.1)	1.0 (0.7, 1.3)	0.6 (0.4, 0.8)
Persons (*n* ^a^), 95% CI	1,068,938 (961,741, 1,176,135)	1,320,437 (1,224,581, 1,416,292)	153,808 (105,319, 202,297)	128,829 (92,774, 164,884)
Frequency of cannabis use in the past 12‐months				
4–7 days per week (%), 95% CI	13.4 (9.4, 18.8)	14.7 (12.0, 17.9)	37.3 (22.7, 54.6)	42.6 (31.9, 54.0)
1–4 days per week (%), 95% CI	23.1 (19.6, 27.1)	17.0 (14.2, 20.4)	37.2 (26.5, 49.3)	24.1 (15.0, 36.3)
Less than once a week (%), 95% CI	63.5 (57.5, 69.1)	68.3 (64.3, 72.0)	25.5 (14.0, 42.0)	33.3 (22.4, 46.4)
Amount of cannabis used on a typical day				
Mean (grams), 95% CI	3.3 (2.5, 4.2)	2.6 (2.3, 2.9)	6.9 (2.9, 10.9)	5.5 (4.2, 6.8)
Median (grams), IQR range	2 (1–4)	1 (1–2)	5 (3–10)	3 (1–10)

Abbreviations: CI, confidence interval; DSM‐IV, Diagnostic and Statistical Manual of Mental Disorders 4th edition; IQR, interquartile range.

^a^ Estimated number of individuals in Australia; survey total sample sizes were 8841 in 2007 and 15,893 in 2022–23.

#### 3.1.2 Past 12‐Month CUD

The prevalence of past 12‐month CUD in 2020–22 was 0.6% (95% CI 0.4%, 0.8%; equivalent to 128,829 Australians), which was stable from 2007 given overlapping CIs (1.0% 95% CI 0.7%, 1.3%; Table 1). Among those reporting a CUD in 2020–22, 33.3% (95% CI 22.4%, 46.4%) reported using cannabis less than once a week, 24.1% (95% CI 15.0%, 36.3%) used 1–4 days per week and 42.6% (95% CI 31.9%, 54.0%) used 4–7 days per week. This distribution of frequency of use was similar between 2007 and 2020–22, with overlapping CIs. In the same sample, an average of 5.49 g (95% CI 4.19, 6.79) and a median of 3 g (interquartile range 1–10), of cannabis were consumed on a typical day in 2020–22. The amount of cannabis was unchanged between 2007 and 2020–22.

### 3.2 Association Between Individual Characteristics and Cannabis Use Outcomes

#### 3.2.1 Demographics

##### 3.2.1.1 Past 12‐Month Cannabis Use

**FIGURE 1:**
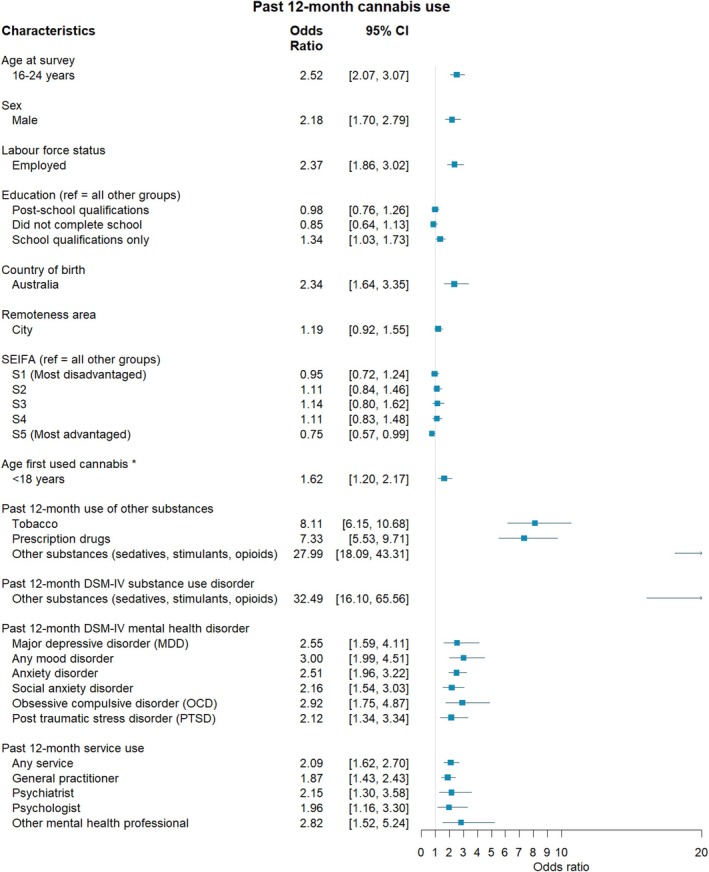
The association between individual characteristics and past 12‐month cannabis use among the combined 2007 and 2020–22 sample. *Controlling for age at survey.

**FIGURE 2:**
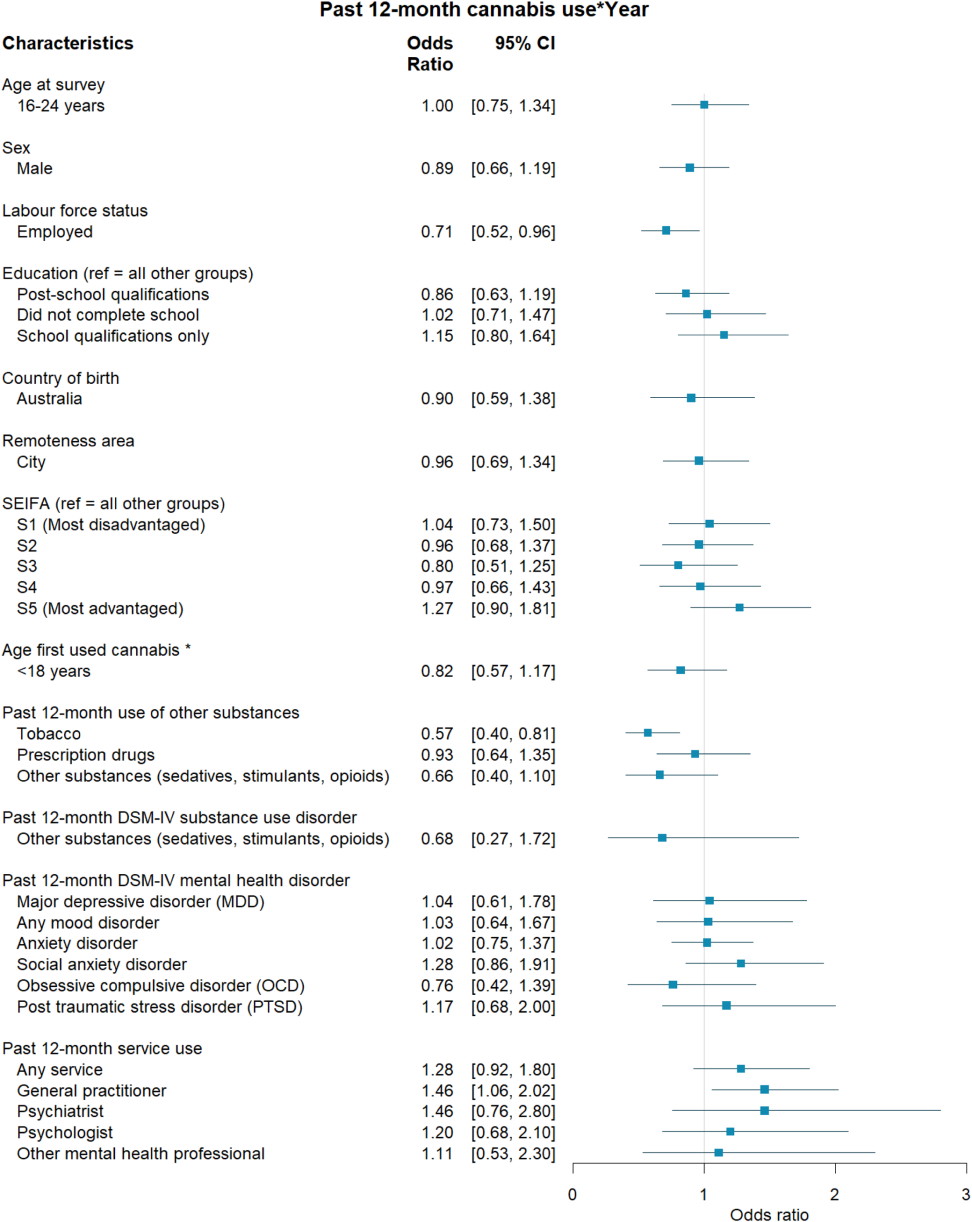
The association between individual characteristics and past 12‐month cannabis use according to year of survey. *Note:* Odds ratios and 95% CI above 1 represent an increase in the strength of a relationship from 2007 to 2020–22. < 1 = a decrease in the strength of the relationship from 2007 to 2020–22. *Controlling for age at survey.

Table S1 shows the proportion of those reporting cannabis use according to demographic, substance use, mental health and health service use characteristics. Regression analysis of the combined 2007 and 2020–22 sample revealed significantly higher odds of cannabis use among those aged 16–25 years compared to those 26 years and above (OR = 2.52, 95% CI 2.07, 3.07) and among males compared to females (OR = 2.18, 95% CI 1.70, 2.79; Figure 1). Respondents who were employed demonstrated significantly higher odds of cannabis use compared to those that were unemployed or not in the workforce (OR = 2.37, 95% CI 1.86, 3.02), but this relationship weakened from 2007 to 2020–22 (OR = 0.71, 95% CI 0.52, 0.96; Figure 2, also see Figure S1). As for education, those with school qualifications only had higher odds of cannabis use compared to both non‐school completers and those with post‐school qualifications (OR = 1.34, 95% CI 1.03, 1.73). Australian‐born respondents had significantly higher odds of cannabis use compared to those born outside of Australia (OR = 2.34, 95% CI 1.64, 3.35). Lastly, those with the lowest Index of Relative Socioeconomic Disadvantage (most advantaged) had significantly higher odds of cannabis use compared to those with higher levels of disadvantage (OR = 0.75, 95% CI 0.57, 0.99).

##### 3.2.1.2 Past 12‐Month CUD

**FIGURE 3:**
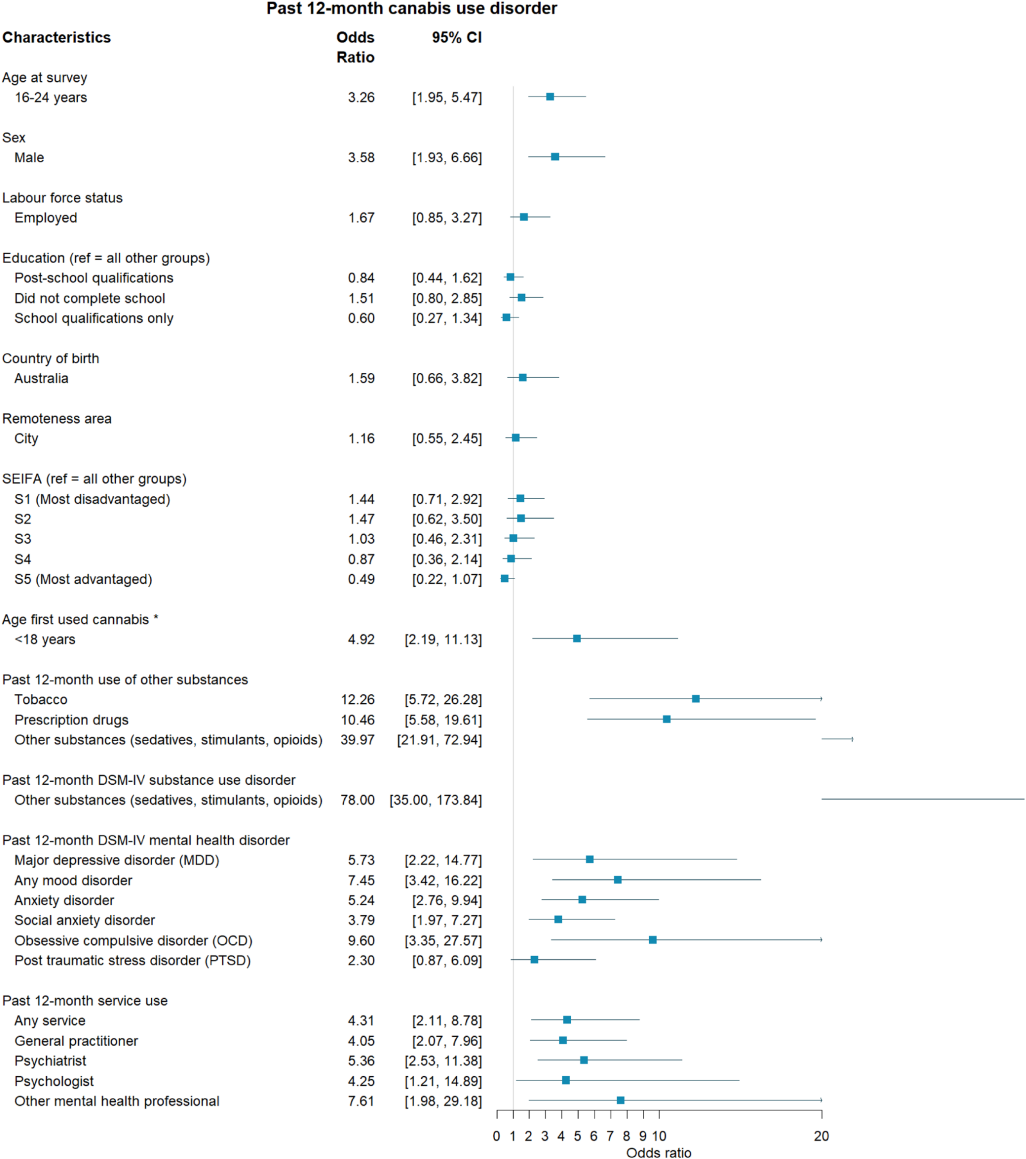
The association between individual characteristics and past 12‐month cannabis use disorder among the combined 2007 and 2020–22 sample. *Controlling for age at survey.

**FIGURE 4:**
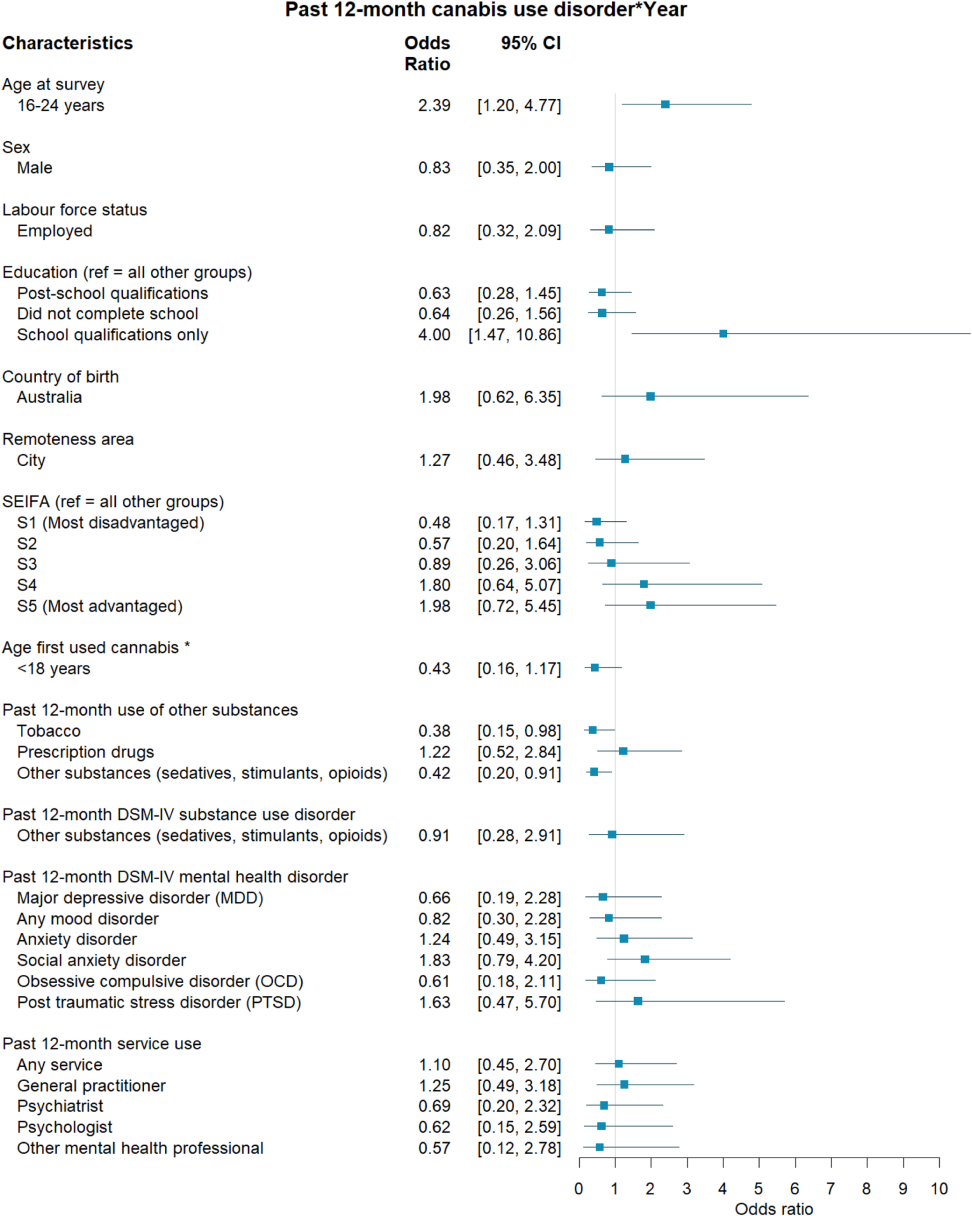
The association between individual characteristics and past 12‐month cannabis use disorder according to year of survey. *Note:* Odds ratios and 95% CI above 1 represent an increase in the strength of a relationship from 2007 to 2020–22. < 1 = a decrease in the strength of the relationship from 2007 to 2020–22. *Controlling for age at survey.

Table S1 shows the proportion of those reporting cannabis use according to demographic, substance use, mental health and health service use characteristics. Among the combined sample, those aged 16–25 years had greater odds of reporting a CUD compared to those 26 years and older (OR = 3.26, 95% CI 1.95, 5.47; Figure 3). When including year of survey as an interaction, the relationship between younger respondents' age and CUD became stronger from 2007 to 2020–22 (OR = 2.39, 95% CI 1.20, 4.77; Figure 4, also see Figure S2). While there was no main effect between school qualifications only (regardless of year) and the odds of CUD, those with school qualifications only had greater odds of reporting a CUD in 2020–22 relative to 2007 (OR = 4.00, 95% CI 1.47, 10.86; Figure S3).

#### 3.2.2 Polysubstance Use

##### 3.2.2.1 Past 12‐Month Cannabis Use

Among the combined sample, after controlling for age at survey, respondents had significantly greater odds of reporting cannabis use when their age of first use was before 18‐years (OR = 1.62, 95% CI 1.20, 2.17; Figure 1). Past 12‐month use of tobacco was associated with a significantly greater likelihood of reporting cannabis use (OR = 8.11, 95% CI 6.15, 10.68), but this relationship weakened from 2007 to 2020–22 (OR = 0.57, 95% CI 0.40, 0.81; Figure 2, also see Figure S4). Those reporting past 12‐month use of prescription drugs (OR = 7.33, 95% CI 5.53, 9.71) and other substances (i.e., sedatives, stimulants, or opioids; OR = 27.99, 95% CI 18.09, 43.31) had significantly greater odds of reporting cannabis use.

##### 3.2.2.2 Past 12‐Month CUD

Among the combined sample, after controlling for age at survey, respondents had significantly greater odds of reporting a CUD when their age of first use was before 18 years of age (OR = 4.94, 95% CI 2.19, 11.13; Figure 3). Those that reported past 12‐month use of tobacco (OR = 12.26, 95% CI 5.72, 26.28), prescription drugs (OR = 10.46, 95% CI 5.58, 19.61) and other substances (OR = 39.97, 95% CI 21.92, 72.94) had greater odds of reporting a CUD. However, the relationship between use of tobacco (OR = 0.38, 95% CI 0.15, 0.98, also see Figure S5), other substances (OR = 0.42, 95% CI 0.20, 0.91, Figure S6) and CUD weakened from 2007 to 2020–22 (Figure 4).

#### 3.2.3 Co‐Occurring Substance Use and Mental Health Disorders

##### 3.2.3.1 Past 12‐Month Cannabis Use

Among the combined sample, persons who reported cannabis use in the past year were more likely to report a past 12‐month DSM‐IV other substance use disorder (OR = 32.49, 95% CI 16.10, 65.56), major depressive disorder (OR = 2.55, 95% CI 1.59, 4.11), any mood disorder (OR = 3.00, 95% CI 1.99, 4.51), anxiety disorder (OR = 2.51, 95% CI 1.96, 3.22), social anxiety disorder (OR = 2.16, 95% CI 1.54, 3.03), obsessive compulsive disorder (OR = 2.92, 95% CI 1.75, 4.87) and post‐traumatic stress disorder (OR = 2.12, 95% CI 1.34, 3.34) (Figure 1). There were no differences in these associations from 2007 to 2020–22.

##### 3.2.3.2 Past 12‐Month CUD

Among the combined sample, persons were more likely to have a CUD if in the past year they had a DSM‐IV other substance use disorder (OR = 78.00, 95% CI 35.00, 173.84), major depressive disorder (OR = 5.73, 95% CI 2.22, 14.77), any mood disorder (OR = 7.45, 95% CI 3.42, 16.22), anxiety disorder (OR = 5.24, 95% CI 2.76, 9.94), social anxiety disorder (OR = 3.79, 95% CI 1.97, 7.27) and obsessive compulsive disorder (OR = 9.60, 95% CI 3.35, 27.57) (Figure 3). There were no differences in these associations from 2007 to 2020–22.

#### 3.2.4 Health Service Use

##### 3.2.4.1 Past 12‐Month Cannabis Use

Among the combined sample, persons who reported cannabis use in the past year were more likely to report any use in the past 12 months of a health service (OR = 2.09, 95% CI 1.62, 2.70), general practitioner (OR = 1.87, 95% CI 1.43, 2.43), psychiatrist (OR = 2.15, 95% CI 1.30, 3.58), psychologist (OR = 1.96, 95% CI 1.16, 3.30) and other mental health professional (OR = 2.82, 95% CI 1.52, 5.24) (Figure 1). The relationship between past 12‐month use of a general practitioner and cannabis use strengthened from 2007 to 2020–22 (OR = 1.46, 95% CI 1.06, 2.02; Figure 2, also see Figure S7).

##### 3.2.4.2 Past 12‐Month CUD

Among the combined sample, persons who had a CUD were more likely to report the use of any health service (OR = 4.31, 95% CI 2.11, 8.78), general practitioner (OR = 4.05, 95% CI 2.07, 7.96), psychiatrist (OR = 5.36, 95% CI 2.53, 11.38), psychologist (OR = 4.25, 95% CI 1.21, 14.89) and other mental health professional (OR = 7.61, 95% CI 1.98, 29.18) in the past 12 months (Figure 3). There were no differences in these associations from 2007 to 2020–22.

## 4 Discussion

The NSMHWB represents the largest snapshot of mental health and cannabis use in Australia. In 2020–22, 6.7% (95% CI 6.2%, 7.1%) of Australians aged 16–85‐years were estimated to have used cannabis in the past 12‐months, equivalent to 1,320,437 individuals. This prevalence remained stable since 2007. Prevalence of past 12‐month CUD in 2020–22 was 0.6% (95% CI 0.4%, 0.8%), which was also stable since 2007 (1.0%). Cannabis use and CUD were both positively associated with adverse health outcomes such as other substance use and mental health disorders and this relationship was largely consistent over time. From 2007 to 2020–22, there was a decrease in the strength of the relationship between being employed, using tobacco, using other substances and cannabis use outcomes. Whereas there was an increase in the strength of the relationship between younger age at survey, having school qualifications only, seeing a general practitioner and cannabis use outcomes.

The rate of past 12‐month cannabis use and CUD was notably lower than that reported in other Australian estimates. According to the National Drug Strategy Household Survey (NDSHS), 11.5% of Australians reported annual use while 2.2% exhibited a moderate‐ to high‐risk for cannabis use related problems. However, unlike the WHM‐CIDI 3.0 used in the current survey, the National Drug Strategy Household Survey assessed risk using a short version of the Alcohol, Smoking and Substance Involvement Screening Test (ASSIST‐LITE) where moderate‐risk does not necessarily reflect a cut‐off point for DSM‐IV CUD [23]. There are other differences in survey designs making it difficult to compare prevalence estimates, e.g., respondents in the NSMHWB were required to report at‐least 5 past occasions of cannabis consumption. Without this methodological restriction, the prevalence of any cannabis use is likely larger than what is reported in the NSMHWB data. This may also explain why the prevalence of any cannabis use in Australia was less than a third of what was observed in the US (National Survey on Drug Use and Health (NSDUH); 2022–23; 21.8%) and similar to the average recorded in Europe [6, 24].

Unlike the US, there does not appear to be an increase in cannabis use and CUD since 2007 in Australia. If increasing trends of cannabis use observed in the US over time are due to more liberal cannabis policies, this may not be reflected in Australia, where recreational cannabis is still illegal in most states and territories. Furthermore, while medicinal cannabis laws were permitted in 2016, increased uptake occurred from 2019 [25], thus its effects may not have been captured in the recent survey. If Australia is to adopt more permissive cannabis policies, it is critical that we continue to monitor the prevalence of cannabis use, CUD and other adverse consequences.

There were several demographic factors that were associated with both cannabis use and CUD. Those aged 16–25 years and male had twice as greater odds of reporting use, and over three‐times greater odds of reporting a CUD. A similar relationship was seen among those reporting first‐time use prior to 18‐years of age. There is now consistent evidence globally that males who initiate cannabis use at a younger age are more likely to become regular users [26]. These associations could be explained by greater risk‐taking behaviour in youth and individual factors (e.g., sensitivity to psychoactive affects) [27]. Furthermore, prefrontal‐striatal brain pathways that are implicated in the neurobiology of cannabis use are still undergoing significant maturing through youth until age 26 [28] Adolescents may be even more vulnerable to the adverse effects of early use as THC concentrations in cannabis increase [16, 29]. This could explain why CUD has become more common in young people since 2007. Reflecting on these results, it is critical that governments further invest in prevention, harm reduction and treatment strategies that target our youth to reduce the burden of the disease associated with cannabis consumption and CUD.

In line with previous evidence, polysubstance and polysubstance use disorders are common among those using cannabis and with a CUD [30]. Despite a wealth of literature investigating both concurrent polysubstance use [31] and stages of substance use [32], it remains unclear whether this association is due to shared environmental factors or causal mechanisms [33]. It may not be surprising that the association between cannabis use and tobacco use has become less salient since 2007 given that tobacco prevalence in Australia has almost halved since then (19%) [34]. As for the weakened association between cannabis use and other substances, young people may be more aware of the harms associated with substances such as opioids and stimulants. It is also important to note that these sub‐groups have low numbers, as represented by high ORs (39.97) and wide confidence intervals around them, that the model may be susceptible to slight changes in the sample. As such, our findings surrounding this should be interpreted with caution.

Our findings on co‐occurring mental health disorders add to the evidence that cannabis use and CUD may be associated with adverse mental health outcomes. Unfortunately, because the data are cross‐sectional we are unable to draw strong inferences about the nature of the relationship, such as the order of onset of different drugs, or whether the relationships are causal. Among studies that have attempted to control for various confounding factors and order of onset, the strongest relationship is with psychotic disorders, but the evidence is less clear for mood or anxiety disorders, post‐traumatic stress disorder, or obsessive compulsive disorder [35, 36]. Nevertheless, the disproportionate rate of cannabis use and CUD among those with mental health conditions in our sample may also explain why they are more likely to have recently accessed services for their mental health. Unfortunately, the siloed approach to mental health and substance use treatment in Australia may mean that among those seeking treatment services, cannabis use is neglected [37]. It is therefore essential that health workers are equipped with the necessary training to manage co‐occurring mental health and substance use disorders [38].

### 4.1 Limitations

There are several limitations to this study that need to be highlighted. Firstly, considering that this is a cross‐sectional survey, we are unable to infer causality between associates and cannabis use outcomes. Also, without conducting multivariable regression models, we have limited understanding of potential confounding or collinearity among predictors. Secondly, the survey received a response rate of approximately 52%, had limited representation from indigenous communities or remote locations and did not include homeless or currently incarcerated people. Although this raises some doubt to the generalisability of the survey, appropriate weightings were applied to ensure that the sample otherwise resembled the distribution in the population. Furthermore, similar response rates are seen in population surveys internationally [39]. Thirdly, there were a few measures of interest that we could not include in the analysis. As previously mentioned, there is considerable evidence for an association between psychotic disorders and cannabis use [36]. Although these disorders effect a small proportion of people in Australia, they can have a devastating impact on quality of life and the public health system [40]. Psychotic disorders were not included in the survey because they are difficult to assess and there would be few persons in the survey. Lastly, we did not consider the prevalence and associates of CUD severity, which may vary significantly from endorsing 2 (mild) to 11 (severe) symptoms. Recent US national survey data found that 54.4% of people reporting a CUD had a mild disorder [6], a portion of people that may not exhibit chronic patterns of use. This may be reflected in our frequency data, where one‐third of those with a CUD reported using cannabis less than once a week, figures that have been similarly reported elsewhere [41].

## 5 Conclusion

Prevalence of cannabis use and CUD have remained stable among the general Australian population from 2007 to 2020–22. Younger people are more likely to develop CUD than older people (+25‐years), a relationship that has strengthened over time, highlighting the need for effective prevention, treatment and harm reduction strategies. Furthermore, polysubstance use or use disorder was common, and CUD continues to co‐occur with other mental health conditions. As cannabis products become more accessible, it is crucial that we monitor trends in use and develop a public health response to assist those most at risk of harm.

## Author Contributions

**Jack Wilson:** conceptualisation, methodology, software validation, formal analysis, writing – review and editing. **Matthew Sunderland:** methodology, Software validation, Formal analysis, writing – review and editing. **Siobhan O’Dean:** methodology, Visualisation, Formal analysis, writing – review and editing. **Tim Slade:** methodology, writing – review and editing. **Danielle Dawson, Olivia Dobson, Janni Leung, Gary Chan, Maree Teesson, Wayne Hall, Nicola Newton, Valentina Lorenzetti:** writing – review and editing. **Emily Stockings:** conceptualisation, methodology, supervision, funding acquisition.

## Funding

JW and ES are supported by a National Health and Medical Research Council (NHMRC) investigator grant awarded to ES (GNT2017346). JL and GC are supported by NHMRC fellowships. VL is supported by a NHMRC Investigator Grant and by an AI and Val Rosenstrauss Research Fellowship, Rebecca Cooper Foundation.

## Conflicts of Interest

The authors declare no conflicts of interest.

## Supporting information


**Figure S1:** Predicted probability of past 12‐month cannabis use among those employed according to year of survey.
**Figure S2:** Predicted probability of past 12‐month cannabis use disorder among those aged 16–25 years old according to year of survey.
**Figure S3:** Predicted probability of past 12‐month cannabis use disorder among those reporting school qualifications only according to year of survey.
**Figure S4:** Predicted probability of past 12‐month cannabis use among those reporting past 12‐month tobacco use according to year of survey.
**Figure S5:** Predicted probability of past 12‐month cannabis use disorder among those reporting past 12‐month tobacco use according to year of survey.
**Figure S6:** Predicted probability of past 12‐month cannabis use disorder among those reporting past 12‐month other substance use (sedatives, stimulants, or opioids) according to year of survey.
**Figure S7:** Predicted probability of past 12‐month cannabis use among those reporting a past 12‐month visit to a general practitioner according to year of survey.

**Table S1:** Prevalence of cannabis use and use disorder according to individual characteristics.

## Data Availability

The data that support the findings of this study are available from Australian Bureau of Statistics. Restrictions apply to the availability of these data, which were used under license for this study. Data are available from the author(s) with the permission of Australian Bureau of Statistics.
