# Supplementary material for: Prevalence, Associations and Comorbidity of Cannabis Use and Cannabis Use Disorders in the Australian National Mental Health Surveys From 2007 to 2020–22
Source: Drug Alcohol Rev. 2026 Mar 8;45(3):e70134. doi: 10.1111/dar.70134 (PMC12968346; doi:10.1111/dar.70134)

Figure S1. Predicted probability of past 12-month cannabis use among those employed according to year of survey


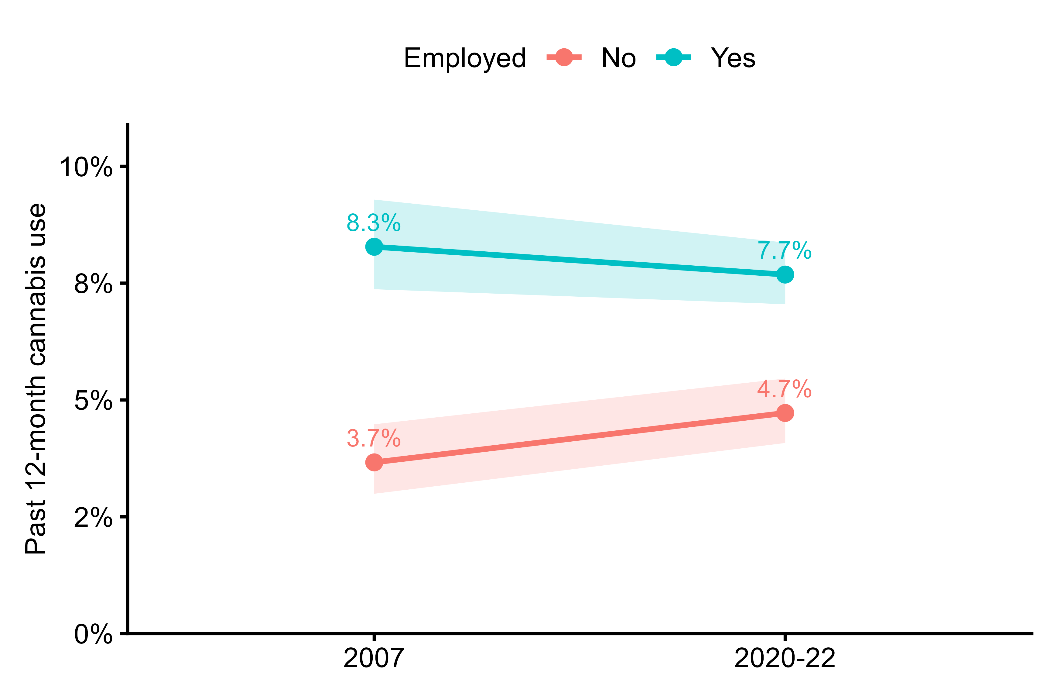


Figure S2. Predicted probability of past 12-month cannabis use disorder among those aged 16-25 years old according to year of survey


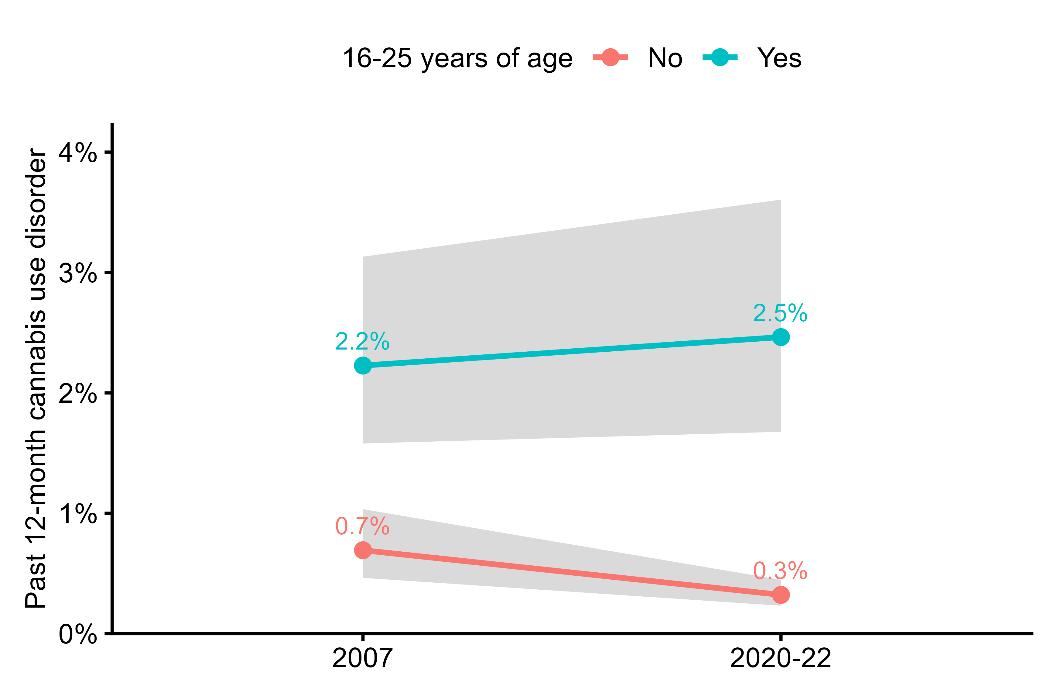


Figure S3. Predicted probability of past 12-month cannabis use disorder among those reporting school qualifications only according to year of survey


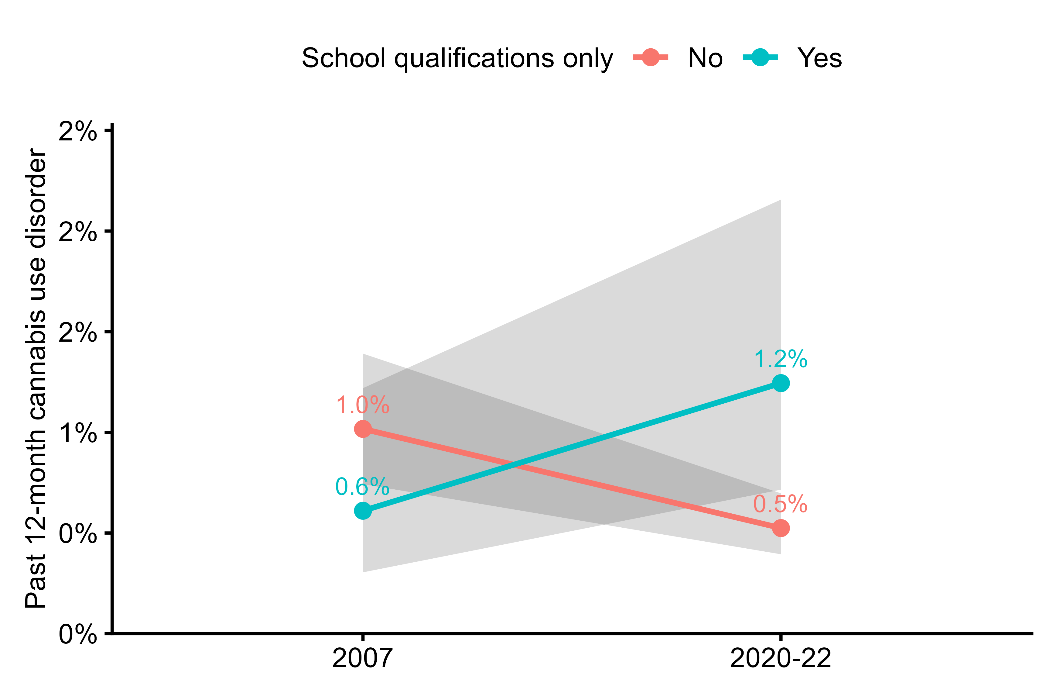


Figure S4. Predicted probability of past 12-month cannabis use among those reporting past 12-month tobacco use according to year of survey


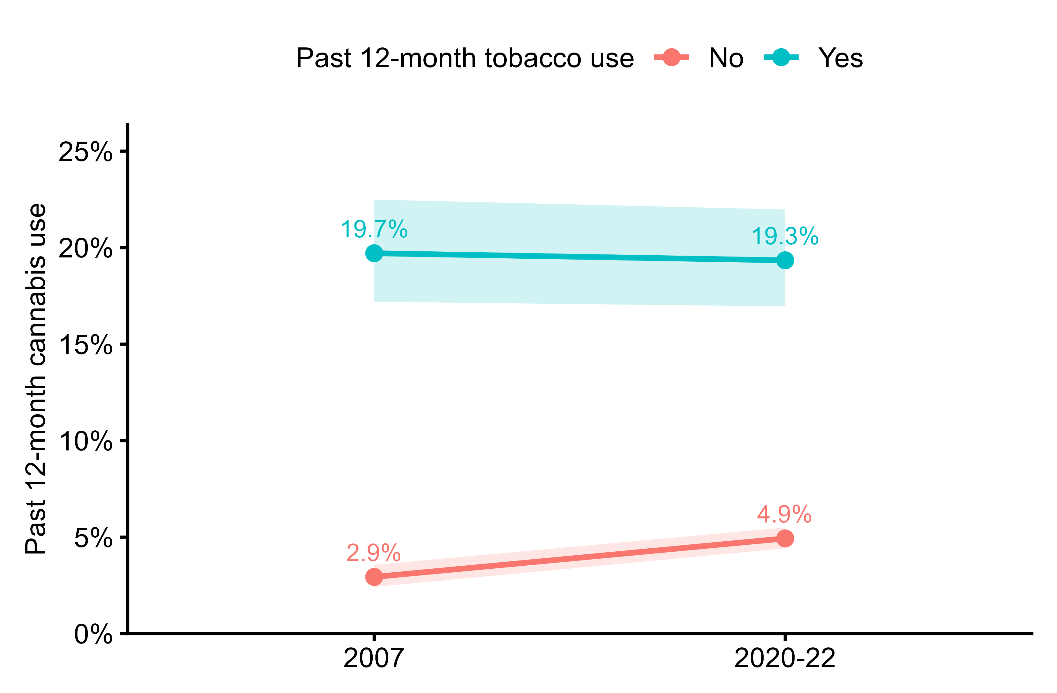


Figure S5. Predicted probability of past 12-month cannabis use disorder among those reporting past 12-month tobacco use according to year of survey


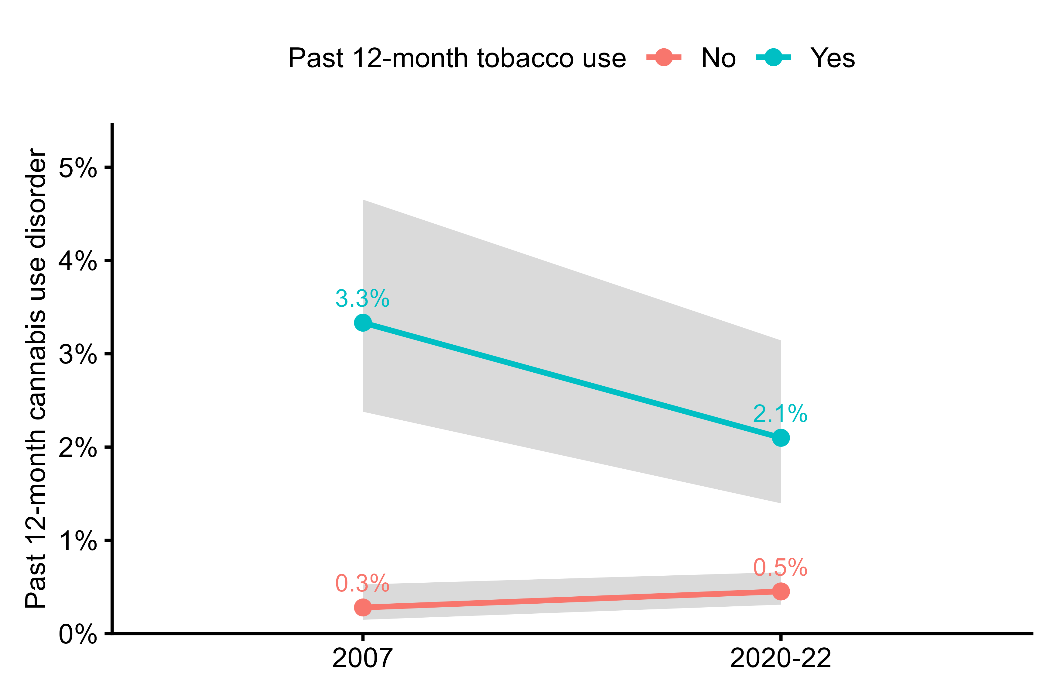


Figure S6. Predicted probability of past 12-month cannabis use disorder among those reporting past 12-month other substance use (sedatives, stimulants, or opioids) according to year of survey


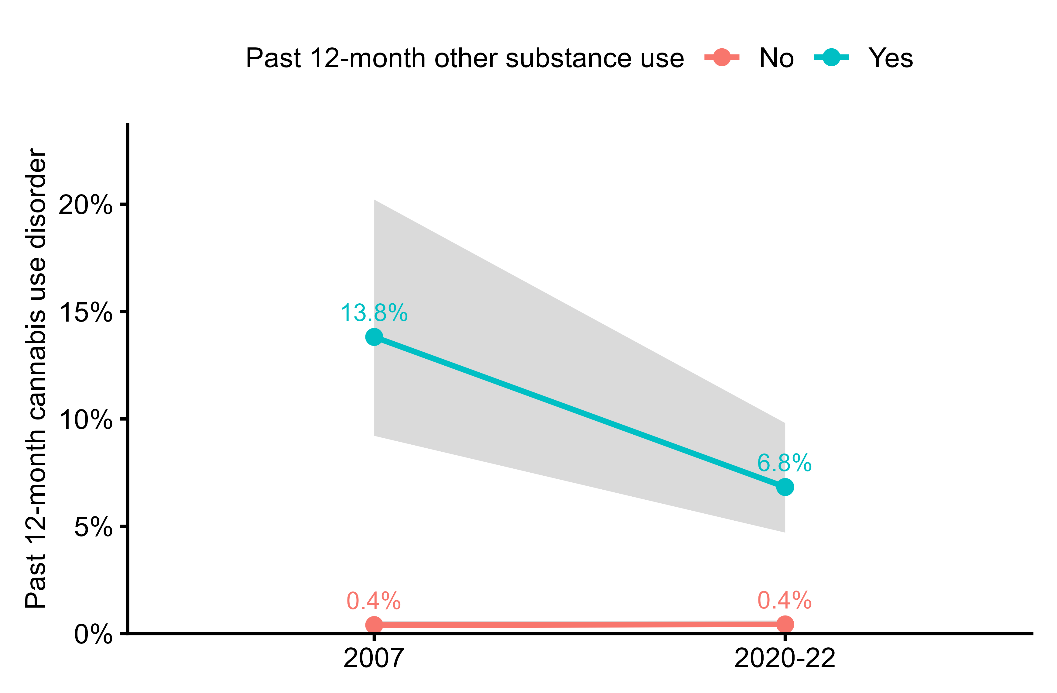


Figure S7. Predicted probability of past 12-month cannabis use among those reporting a past 12-month visit to a general practitioner according to year of survey


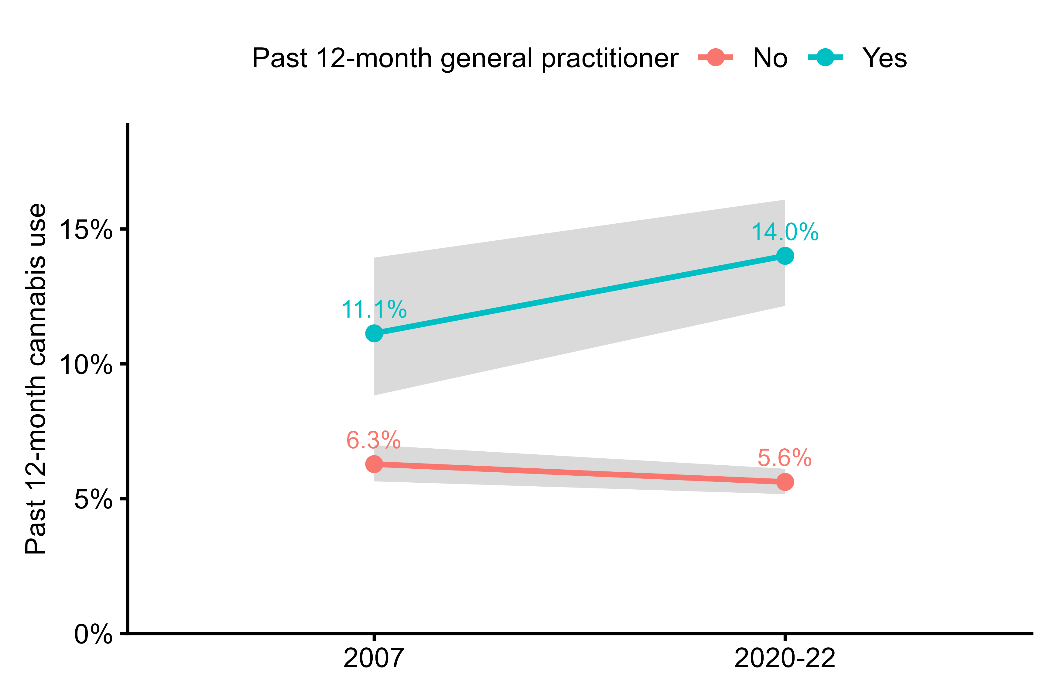

Supplement: Supplementary file 1 — Figure S1: Predicted probability of past 12‐month cannabis use among those employed according to year of survey. Figure S2: Predicted probability of past 12‐month cannabis use disorder among those aged 16–25 years old according to year of survey. Figure S3: Predicted probability of past 12‐month cannabis use disorder among those reporting school qualifications only according to year of survey. Figure S4: Predicted probability of past 12‐month cannabis use among those reporting past 12‐month tobacco use according to year of survey. Figure S5: Predicted probability of past 12‐month cannabis use disorder among those reporting past 12‐month tobacco use according to year of survey. Figure S6: Predicted probability of past 12‐month cannabis use disorder among those reporting past 12‐month other substance use (sedatives, stimulants, or opioids) according to year of survey. Figure S7: Predicted probability of past 12‐month cannabis use among those reporting a past 12‐month visit to a general practitioner according to year of survey. [file DAR-45-0-s002.docx]
