# Supplementary material for: Prevalence, Associations and Comorbidity of Cannabis Use and Cannabis Use Disorders in the Australian National Mental Health Surveys From 2007 to 2020–22
Source: Drug Alcohol Rev. 2026 Mar 8;45(3):e70134. doi: 10.1111/dar.70134 (PMC12968346; doi:10.1111/dar.70134)
Supplement: Supplementary file 2 — Table S1: Prevalence of cannabis use and use disorder according to individual characteristics. [file DAR-45-0-s001.docx]

Table S1. Prevalence of cannabis use and use disorder according to individual characteristics

|  | **Past 12-month cannabis use** | | **Past 12-month DSM-IV any cannabis use disorder** | |
| --- | --- | --- | --- | --- |
|  | **2007** | **2023** | **2007** | **2023** |
| **Age at survey** | | | | |
| 16-25 (%)  *95% CI* | 12.6%  *(10.7%, 14.9%)* | 12.9%  *(11.3%, 14.7%)* | 2.2%  *(1.4%, 3.6%)* | 2.5%  *(1.7%, 3.6%)* |
| ≥26 (%)  *95% CI* | 5.4%  *(4.7%, 6.2%)* | 5.5%  *(5.0%, 6.1%)* | 0.7%  *(0.4%, 1.1%)* | 0.3%  *(0.2%, 0.4%)* |
| **Sex** | | | | |
| Male (%)  *95% CI* | 9.0%  *(8.0%, 10.1%)* | 8.7%  *(7.9%, 9.5%)* | 1.5%  *(1.1%, 2.1%)* | 1.0%  *(0.7%, 1.4%)* |
| Female (%)  *95% CI* | 4.4%  *(3.6%, 5.2%)* | 4.7%  *(4.2%, 5.3%)* | 0.4%  *(0.2%, 0.8%)* | 0.3%  *(0.2%, 0.5%)* |
| **Labour force status** | | | | |
| Employed (%)  *95% CI* | 8.3%  *(7.4%, 9.2%)* | 7.7%  *(7.1%, 8.4%)* | 1.1%  *(0.7%, 1.7%)* | 0.7%  *(0.5%, 1.0%)* |
| Unemployed /not in the labour force (%)  *95% CI* | 3.7%  *(3.0%, 4.5%)* | 3.9%  *(3.3%, 4.7%)* | 0.7%  *(0.4%, 1.0%)* | 0.2%  *(0.1%, 0.5%)* |
| **Education** | | | | |
| Post-school qualification (%)  *95% CI* | 6.6%  *(5.8%, 7.6%)* | 6.2%  *(5.6%, 6.9%)* | 0.9%  *(0.5%, 1.5%)* | 0.5%  *(0.3%, 0.7%)* |
| School qualification only (%)  *95% CI* | 8.4%  *(6.8%, 10.2%)* | 9.1%  *(7.6%,10.8%)* | 0.6%  *(0.3%, 1.5%)* | 1.2%  *(0.7%, 2.1%)* |
| Did not complete school (%)  *95% CI* | 6.0%  *(4.9%, 7.3%)* | 6.0%  *(4.9%, 7.3%)* | 1.2%  *(0.8%, 1.9%)* | 0.6%  *(0.4%, 1.1%)* |
| **Country of birth** | | | | |
| Australia (%)  *95% CI* | 7.9%  *(7.1%, 8.7%)* | 8.0%  *(7.4%, 8.7%)* | 1.1%  *(0.7%, 1.5%)* | 0.8%  *(0.6%, 1.1%)* |
| **Remoteness area** | | | | |
| Major cities (%)  *95% CI* | 7.0%  *(6.3%, 7.9%)* | 6.9%  *(6.3%, 7.4%)* | 1.0%  *(0.7%, 1.4%)* | 0.7%  *(0.5%, 1.0%)* |
| Inner regional/outer regional/remote/very remote (%)  *95% CI* | 6.0%  *(4.9%- 7.3%)* | 6.0%  *(5.1%- 7.2%)* | 0.9%  *(0.4%- 1.9%)* | 0.5%  (*0.3%- 0.8%)* |
| **SEIFA (Index of Relative Socioeconomic Disadvantage)** | | | | |
| 1 (Most disadvantaged) (%)  *95% CI* | 6.4%  *(5.2%, 7.8%)* | 6.6%  *(5.4%, 8.1%)* | 1.3%  *(0.8%, 2.2%)* | 0.5%  *(0.3%, 0.8%)* |
| 2 (%)  *95% CI* | 7.2%  *(5.9%, 8.8%)* | 7.0%  *(5.8%, 8.4%)* | 1.3%  *(0.7%, 2.4%)* | 0.6%  *(0.3%, 1.0%)* |
| 3 (%)  *95% CI* | 7.3%  *(5.8%, 9.3%)* | 6.2%  *(5.0%, 7.6%)* | 1.0%  *(0.4%, 2.4%)* | 0.6%  *(0.3%, 1.3%)* |
| 4 (%)  *95% CI* | 7.2%  *(5.9%, 8.7%)* | 7.0%  *(5.8%, 8.5%)* | 0.9%  *(0.6%, 1.2%)* | 0.9%  *(0.6%, 1.4%)* |
| 5 (Most advantaged) (%)  *95% CI* | 5.4%  *(4.1%, 7.1%)* | 6.4%  *(5.5%, 7.5%)* | 0.5%  *(0.3%, 1.0%)* | 0.6%  *(0.4%, 1.1%)* |
| **Age first time used cannabis (years)** | | | | |
| <18 (%)  *95% CI* | 41.9%  *(37.8%, 46.1%)* | 36.8%  *(33.7%, 40.1%)* | 7.6%  *(5.7%, 9.9%)* | 4.3%  *(3.2%, 5.8%)* |
| 18≥ (%)  *95% CI* | 23.4%  *(18.5%, 29.1%)* | 27.3%  *(24.7%, 30.1%)* | 1.0%  *(0.1%, 8.2%)* | 1.7%  *(1.0%, 2.8%)* |
| **Past 12-month use of other substances** | | | | |
| Tobacco (%)  *95% CI* | 19.7%  *(17.4%, 22.3%)* | 19.3%  *(17.0%, 22.0%)* | 3.3%  *(2.2%, 4.9%)* | 2.1%  *(1.4%, 3.1%)* |
| Non-medical prescription drugs (%)  *95% CI* | 27.5%  *(22.7%, 32.8%)* | 26.8%  *(23.4%, 30.5%)* | 5.6%  *(3.0%, 10.2%)* | 4.4%  *(2.9%, 6.5%)* |
| Other substances (sedatives, stimulants, and opioids) (%)  *95% CI* | 56.8%  *(48.6%, 64.6%)* | 49.9%  *(43.9%, 56.0%)* | 13.8%  *(8.0%, 22.8%)* | 6.8%  *(4.7%, 9.7%)* |
| **Past 12-month DSM-IV substance use disorder** | | | | |
| Other substances (sedatives, stimulants, and opioids) (%)  *95% CI* | 68.3%  *(66.5%, 70.0%)* | 60.4%  *(59.6%, 61.2%)* | 35.5%  *(33.5%, 37.6%)* | 28.4%  *(27.6%, 29.3%)* |
| **Past 12-month DSM-IV mental disorder** | | | | |
| Major depressive disorder (%)  *95% CI* | 14.6%  *(11.1%, 18.9%)* | 14.8%  *(12.2%, 17.8%)* | 4.4%  *(2.3%, 8.0%)* | 2.1%  *(1.1%, 4.0%)* |
| Any mood disorder (%)  *95% CI* | 16.3%  *(12.8%, 20.4%)* | 16.3%  *(13.6%, 19.4%)* | 5.0%  *(3.0%, 8.3%)* | 2.8%  *(1.7%, 4.8%)* |
| Anxiety disorder (%)  *95% CI* | 13.4%  *(10.5%, 16.9%)* | 12.9%  *(11.6%, 14.4%)* | 3.3%  *(2.1%, 5.1%)* | 2.2%  *(1.5%, 3.3%)* |
| Social anxiety disorder (%)  *95% CI* | 12.9%  *(9.1%, 17.9%)* | 14.9%  *(12.7%, 17.4%)* | 3.2%  *(1.7%, 6.0%)* | 3.0%  *(2.1%, 4.5%)* |
| Obsessive compulsive disorder (%)  *95% CI* | 16.6%  *(11.9% ,22.8%)* | 13.1%  *(10.2%, 16.7%)* | 7.1%  *(4.7%, 10.5%)* | 3.1%  *(1.8%, 5.3%)* |
| Post-traumatic stress disorder (%)  *95% CI* | 12.6%  *(8.4%, 18.5%)* | 14.3%  *(11.5%, 17.7%)* | 2.1%  *(0.9%, 4.7%)* | 2.2%  *(1.1%, 4.3%)* |
| **Past 12-month service use** | | | | |
| Any service (%)  *95% CI* | 11.8%  *(9.8%, 14.1%)* | 13.1%  *(11.2%, 15.2%)* | 2.9%  *(2.0%, 4.3%)* | 1.9%  *(1.3%, 2.7%)* |
| General practitioner (%)  *95% CI* | 11.1%  *(8.9%, 13.8%)* | 14.0%  *(12.2%, 16.1%)* | 3.1%  *(2.1%, 4.5%)* | 2.2%  *(1.4%, 3.4%)* |
| Psychiatrist (%)  *95% CI* | 13.1%  *(9.3%, 18.0%)* | 17.4%  *(12.5%, 23.7%)* | 4.5%  *(2.0%, 10.0%)* | 2.2%  *(1.0%, 4.7%)* |
| Psychologist (%)  *95% CI* | 11.9%  *(8.7%, 16.2%)* | 13.2%  *(11.3%, 15.5%)* | 3.6%  *(2.0%, 6.4%)* | 1.5%  *(0.9%, 2.5%)* |
| Other mental health professional (%)  *95% CI* | 16.4%  *(10.8%, 24.0%)* | 17.4%  *(12.9%, 23.0%)* | 6.2%  *(2.4%, 15.2%)* | 2.5%  *(1.3%, 4.9%)* |

CI, confidence interval; DSM-IV, Diagnostic and Statistical Manual of Mental Disorders 4th edition.
